# Magnetic frustration, short-range correlations and the role of the paramagnetic Fermi surface of PdCrO_2_

**DOI:** 10.1038/srep12428

**Published:** 2015-07-24

**Authors:** David Billington, David Ernsting, Thomas E. Millichamp, Christopher Lester, Stephen B. Dugdale, David Kersh, Jonathan A. Duffy, Sean R. Giblin, Jonathan W. Taylor, Pascal Manuel, Dmitry D. Khalyavin, Hiroshi Takatsu

**Affiliations:** 1 H.H. Wills Physics Laboratory, University of Bristol, Tyndall Avenue, Bristol, BS8 1TL, United Kingdom; 2 Department of Physics, University of Warwick, Coventry, CV4 7AL, United Kingdom; 3 School of Physics and Astronomy, Cardiff University, Queen’s Building, The Parade, Cardiff, CF24 3AA, United Kingdom; 4 DMSC - European Spallation Source, Universitetsparken 1, Copenhagen 2100, Denmark; 5 ISIS Facility, Rutherford Appleton Laboratory, Chilton, Didcot, OX11 0QX, United Kingdom; 6 Department of Physics, Tokyo Metropolitan University, Tokyo 192-0397, Japan

## Abstract

Frustrated interactions exist throughout nature, with examples ranging from protein folding through to frustrated magnetic interactions. Whilst magnetic frustration is observed in numerous electrically insulating systems, in metals it is a rare phenomenon. The interplay of itinerant conduction electrons mediating interactions between localised magnetic moments with strong spin-orbit coupling is likely fundamental to these systems. Therefore, knowledge of the precise shape and topology of the Fermi surface is important in any explanation of the magnetic behaviour. PdCrO_2_, a frustrated metallic magnet, offers the opportunity to examine the relationship between magnetic frustration, short-range magnetic order and Fermi surface topology. By mapping the short-range order in reciprocal space and experimentally determining the electronic structure, we have identified the dual role played by the Cr electrons in which the itinerant ones on the nested paramagnetic Fermi surface mediate the frustrated magnetic interactions between local moments.

Frustrated magnetic systems have opened the door to a wide range of fundamentally new exotic behaviour with experimental studies unearthing numerous emergent phenomena^1^,^2^,^3^,^4^. In magnetic crystals, frustration can manifest itself as a competition between localised spins on a lattice interacting through various exchange pathways that cannot be simultaneously satisfied, leading to a large degeneracy of the ground state spin structure. In metals, the presence of conduction electrons means that the exchange interactions are not necessarily dominated by nearest (or next-nearest) neighbours, as they are in insulators and, in frustrated metallic magnets, there is evidence that short-range magnetic correlations exist well above any ordering temperature^5^,^6^,^7^. Spin-orbit physics can be strongly enhanced by electron correlations^8^ and the interactions are thought to result in the large Wilson ratios, observed in many frustrated magnets at low temperatures^9^,^10^.

In this study, we investigate the magnetically frustrated metal PdCrO_2_ and have observed and determined the nature of the short-range magnetic order in the paramagnetic phase through single-crystal neutron diffraction measurements of the diffuse magnetic scattering. In order to determine exactly what role the itinerant conduction electrons play in this frustrated magnet, high-resolution x-ray Compton scattering has been used to determine the topology of the paramagnetic bulk Fermi surface. This has allowed the validation of the calculated electronic structure from which it is shown that the Fermi surface of this system has a propensity for nesting at a wave-vector that is concomitant with the observed short-range magnetic order above the ordering temperature and subsequent magnetic ordering below. Finally, by resolving the crystal momentum-dependence of the nesting instability, we have shown exactly which conduction electrons are responsible for mediating the frustrated magnetic interactions, culminating in the observed magnetic order.

PdCrO_2_ crystallises in the delafossite structure which consists of alternate stacks of a conductive triangular lattice of Pd atoms and a magnetic triangular lattice of edge-shared CrO_6_ octahedra, as shown in Fig. 1a^11^. Below the Néel temperature, *T*_N_ = 37.5 K, the Cr^3+^ (*S* = 3/2) spins order in a commensurate, non-collinear and non-coplanar 120° antiferromagnetic spin structure with 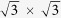 periodicity (the magnetic unit cell is shown in Fig. 1b)^12^,^13^,^14^,^15^. The metallic conductivity shows a strong two-dimensional anisotropy with resistivity measurements revealing a ratio of *c*-axis to *ab*-plane resistivity greater than 150 for all temperatures between 0.32–300 K and greater than 300 at *T* = 0.32 K^11^,^15^,^16^,^17^. In spite of this large anisotropy, the relative magnitude of the drop in the resistivity at *T*_N_ for the *ab*-plane is as large as that for the *c*-axis. The drop in resistivity is attributed to the reduced disorder of the magnetic spins and is associated with the development of short-range spin correlations as *T*_N_ is approached and the long-range antiferromagnetic order below *T*_N_. In the ordered phase, the metallic conductivity is thought to be caused by the delocalised and highly-polarisable Pd 4*d*-electrons^18^,^19^,^20^,^21^. Interestingly, an unconventional anomalous Hall effect has been seen in this compound at temperatures lower than T^*^ = 20 K^22^ and it has recently been reported that a tilting of the 120° spin planes in different Cr layers gives rise to a finite scalar spin chirality which, in the presence of a net magnetisation from an applied magnetic field, may be responsible for the observed effect^14^,^23^. These results indicate substantial coupling between the localised spins within the Cr layers and the conduction electrons in the Pd layer^15^,^21^. The “frustration parameter”, *f*, for magnetic systems is defined as the absolute ratio of the Weiss temperature, Θ_w_, to the ordering temperature, *T*_N_^3^. For PdCrO_2_, *T*_N_ = 37.5 K and Θ_w_ = −500 K giving *f* ≈ 13, illustrating the highly frustrated nature of this system^12^,^13^,^15^. Although there has been a focus on the magnetically ordered phase of PdCrO_2_^13^,^14^,^18^,^19^,^20^,^21^,^22^, much less attention has been paid to the paramagnetic state and, in particular, the region just above *T*_N_.

## Results

### Characterising the short-range magnetic order

We start by experimentally determining the structure and symmetry of the short-range magnetic correlations in reciprocal space through single-crystal neutron diffraction measurements. Figure 2 shows the magnetic scattering in the (*hk*0) and 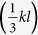 planes determined from single-crystal neutron diffraction at temperatures above and below *T*_N_. Below *T*_N_, magnetic Bragg peaks are seen at 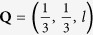 and 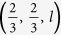 where 
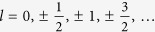
, in agreement with previous powder^13^,^15^ and single-crystal^14^ neutron diffraction studies, which indicated a commensurate non-coplanar 120° spin structure with 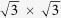 periodicity. The magnetic Bragg peaks are broader along *l* (with a correlation length of 97 ± 6 Å), indicating that the system is inherently less correlated along the *c*-axis. At 38.5 K (1 K above *T*_N_) diffuse magnetic scattering is observed around the location of the magnetic Bragg peaks in the *hk*-plane. The diffuse scattering is quite broad in the *hk*-plane (with correlation lengths of 55 ± 3 Å and 93 ± 5 Å in *h* and *k*, respectively) and extends in rods along *l*, implying that the magnetic correlations are two-dimensional in nature. These diffuse magnetic scattering features are similar to other layered antiferromagnets above their respective ordering temperatures^24^.

In Fig. 2b, there exists a second set of magnetic Bragg peaks originating from a second crystallite that is misaligned from the first by about 5°. In the Compton scattering experiment (discussed below), the beam size was carefully chosen so as to only scatter off one of these crystallites, otherwise we would not have observed meaningful directional anisotropy which would have made a reconstruction of the Fermi surface impossible.

### Electronic structure calculations.

First-principles calculations of the electronic band structure, Fermi surface and density-of-states (DOS) of PdCrO_2_ were performed. The calculated band structure of paramagnetic PdCrO_2_ is shown in Fig. 3a. In this phase, the calculations show that two bands cross the Fermi level, *E*_F_, and the resulting Fermi surface sheets from band 1 and 2 are shown in Fig. 3b,c, respectively. At *E*_F_, both bands have predominantly Cr *d* character, although the first band (band 1, outer sheet) is much more strongly hybridised with Pd *d* than the second (band 2, inner sheet) which is almost completely Cr *d*. Interestingly, the Cr *d*-electron (band 2) sheet has not been seen before in previous photoemission investigations above *T*_N_^18^,^20^ and electronic structure calculations of the paramagnetic phase have not been reported previously, with calculations of a theoretical ferromagnetic phase^21^ (which has not been seen experimentally) being used for comparison in one experimental study^18^. The calculated DOS of paramagnetic PdCrO_2_ is shown in Fig. 3d and the DOS at *E*_F_, N(*E*_F_), is 3.17 states (eV f.u.)^−1^.

Calculations were also performed for 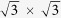 120° non-collinear antiferromagnetic phase of PdCrO_2_ and showed good agreement with previous studies^18^,^19^,^20^. The calculated DOS of this phase is shown in Fig. 3e. Because of the magnetic ordering, the mostly Cr *d*-electron Fermi surface sheet (band 2) becomes fully gapped at *E*_F_ leading to a drop in *N*(*E*_F_). The size of the gap is approximately 2 eV. This leaves only one band crossing *E*_F_ which is then folded into the smaller magnetic Brillouin zone. At *E*_F_, the character of this band is mostly Pd *d*-electron and *N*(*E*_F_) is 0.62 states (eV f.u.)^−1^ which is substantially lower than the paramagnetic phase.

### Fermi surface measurement

Having calculated the electronic structure, we now validate these calculations by measuring the bulk Fermi surface in the paramagnetic phase. Compton scattering is a uniquely powerful probe of the ground-state electronic wave function and directly determines the occupancy of the electron momentum states^25^,^26^. A Compton profile, *J*(*p*_*z*_), is a double integral of the electron momentum distribution, *ρ*(**p**),


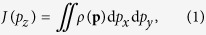


where *p*_*z*_ is taken along the scattering vector and *ρ*(**p**) can be expressed as,


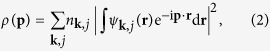


where *ψ*_**k**,*j*_(**r**) is the wave function of the electron in band *j* with wave-vector **k**, and n_**k,**_*_j_* is its occupation.

Five Compton profiles were measured, spaced equally between the Γ-M and Γ-K directions (spanning 30°) of the hexagonal Brillouin zone. The directional differences, Δ*J*(*p*_*z*_), of the five measured Compton profiles relative to the Γ-M direction are plotted in Fig. 4 together with the calculated directional differences for comparison. In order to gauge the sensitivity of Compton scattering to the Fermi surface of this system, the directional difference calculations were performed with all of the bands included and with only the fully occupied bands. Since the fully occupied bands do not cross *E*_F_, they do not contribute to the Fermi surface. Therefore, the improved agreement between experiment and calculation when the bands crossing *E*_F_ are included in the calculation indicates that Compton scattering is extremely sensitive to the Fermi surface of this system.

By applying the Cormack reconstruction method to the Compton profiles^27^, a projected two-dimensional distribution along an axis perpendicular to the set of one-dimensional projections can be recovered. Application of the Lock-Crisp-West technique^28^ then allows us to fold the **p**-space distribution back into the first Brillouin zone in order to give the projected **k**-space occupation density. Since the Fermi surface separates occupied from unoccupied states, it presents itself as a sharp change in the electron occupancy which can be directly visualised in the **k**-space occupation density.

Figure 5 shows the projected occupation density of PdCrO_2_ extracted from the Compton scattering experiment with that predicted by electronic structure calculations. The qualitative agreement between calculation and experiment is excellent. To unambiguously show that both sheets are resolved, a path through the projected occupation density is presented in Fig. 6 in which the Compton data are compared to the predicted contributions of the two separately calculated bands (together with their sum). Here, agreement between experiment and theory is only present when both bands are included, confirming the existence of the two electron-like Fermi surface sheets predicted by the electronic structure calculation (shown superimposed over the bottom right quadrant of Fig. 5). The inclusion of spin-orbit coupling in the calculation was an essential ingredient for such agreement. The occupied fraction of the central hexagonal sheet of band 1 can be estimated from our Compton data as 0.46 ± 0.05, which agrees well with that obtained from photoemission (0.45 ± 0.06)^20^, and the value 0.502 associated with the *δ* (magnetic breakdown, and hence non-magnetic Fermi surface) orbit in the quantum oscillatory study of Ok *et al.*^19^.

### Fermi surface nesting

Having determined the paramagnetic Fermi surface of PdCrO_2_ and shown that the calculated electronic structure provides an excellent description of the experimental data, we now turn to calculating the screening properties of the itinerant electrons in a frustrated metal. For dynamic perturbations with wave-vector **q** and frequency *ω* (such as spin fluctuations), states lying close to the Fermi surface (and, therefore, with the Fermi momentum, **k**_F_) are heavily involved with any electronic screening response and, therefore, the **q**-dependence of the response will depend on the Fermi surface topology. When **q** spans the Fermi surface, the electrons are able to produce a large response^29^ and this effect is maximised when large flat areas of Fermi surface can be mapped onto each other by a single **q**-vector^30^,^31^. When this occurs, the Fermi surface is said to be nested and this effect will tend to promote short-range magnetic correlations at the nesting vector, q^*^. The relevant quantity here is the generalised susceptibility, *χ*(**q**, *ω*). The noninteracting susceptibility in the constant matrix element approximation, *χ*_0_(**q**), is defined as the low frequency (*ω* → 0) limit of,


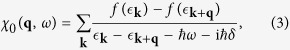


for some perturbation of wave-vector **q** and frequency *ω*. Here, 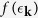 is the Fermi occupancy of the state with energy 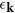 and *δ* is a small time constant for the growth of the perturbation. Since the numerator depends on the occupancies and the denominator depends on the energies of the states **k** and **k** + **q**, this function is maximum when the Fermi surface is nested^30^,^31^. The imaginary part, Im[*χ*_0_(**q**)], is directly related to the Fermi surface topology and is often referred to as the “nesting function”, whilst the real part, Re[*χ*_0_(**q**)], gives the actual screening response of electrons to the perturbation. For an electronic instability, peaks in the imaginary part must carry over into the real part at the same wave-vector^30^.

By decomposing the generalised susceptibility into interband and intraband contributions (which correspond to transitions between the two bands and within the same band, respectively), we have identified nesting in the inner warped hexagonal Fermi surface (band 2) at the wave-vector where the long-range order eventually develops; the interband and intraband contributions from the other sheet do not display this behaviour. Figures 7a,b show the imaginary and real parts, respectively, of *χ*_0_(**q**) in the *q*_*z*_ = 0 plane for intraband transitions of band 2. Both parts peak at 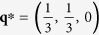 and symmetry related positions, *exactly* where the diffuse magnetic scattering intensity is greatest. We have determined where on the Fermi surface the nesting is occurring by removing the sum in Eq. (3) and calculating *χ*_0,**q**_(**k**) for **q** = q^*^^32^. Here,


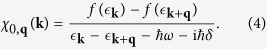


Figure 7c shows the **k**-dependence of the real part of the intraband susceptibility at **q** = q^*^. Here, the “hot spots” indicate electron states which are connected by the nesting vector and therefore contribute to the response. These are located at the rounded corners of the warped hexagonal tube. Interestingly, there is also a significant contribution from states away from *E*_F_ from finite energy transitions^32^. As these are the **k**-states involved in the electronic instability that promotes the formation of spin correlations and subsequent magnetic order, this function connects the Fermi surface topology to the short-range magnetic correlations depicted in Fig. 2. It should be pointed out that a 120° antiferromagnetic spin structure would be supported by a nearest-neighbour antiferromagnetic Heisenberg model on a triangular lattice, and therefore it is clear that Fermi surface nesting is unlikely to be solely responsible for the observed magnetic order. However, the itinerant electrons, owing to the topology of the Fermi surface, are contributing constructively to the observed ordering.

## Discussion

Single-crystal neutron diffraction measurements were performed in order to confirm the previously determined magnetic structure^13^,^14^,^15^ below the ordering temperature and to determine the structure in reciprocal space of the short-range magnetic correlations above the ordering temperature from measurements of the diffuse magnetic scattering. The single-crystal neutron diffraction measurements were complemented by high-resolution x-ray Compton scattering measurements of the electron momentum density in the paramagnetic phase, from which the Fermi surface was inferred from discontinuities in the **k**-space occupation density. The experimentally determined electron momentum density was able to validate the calculated electronic structure and revealed that there are two separate sheets of Fermi surface.

Further calculations of the non-interacting generalised susceptibility revealed that the previously unseen Fermi surface sheet had a propensity for nesting at a wave-vector that is concomitant with the magnetic ordering vector and also where the diffuse magnetic scattering intensity is at its greatest. By resolving the **k**-dependence of the generalised susceptibility, the nested electron states that help promote the short-range magnetic correlations were revealed. Above *T*_N_, the Cr electrons in the nested band partially relieve the frustration through screening of the local exchange interactions thereby promoting the formation of short-range antiferromagnetic correlations at the nesting vector. At *T*_N_, long-range magnetic order starts to appear and the nested band becomes fully gapped leaving only the strongly hybridised band with a redistribution of states and slightly modified band topology, presumably due to the change in crystal field. Below *T*_N_, this band is folded into the smaller magnetic Brillouin zone.

This study constitutes detailed experimental evidence of the correlation between Fermi surface topology and magnetic frustration in metals and provides a benchmark in understanding frustrated metallic magnets.

## Methods

### Crystal growth

**Figure 1:**
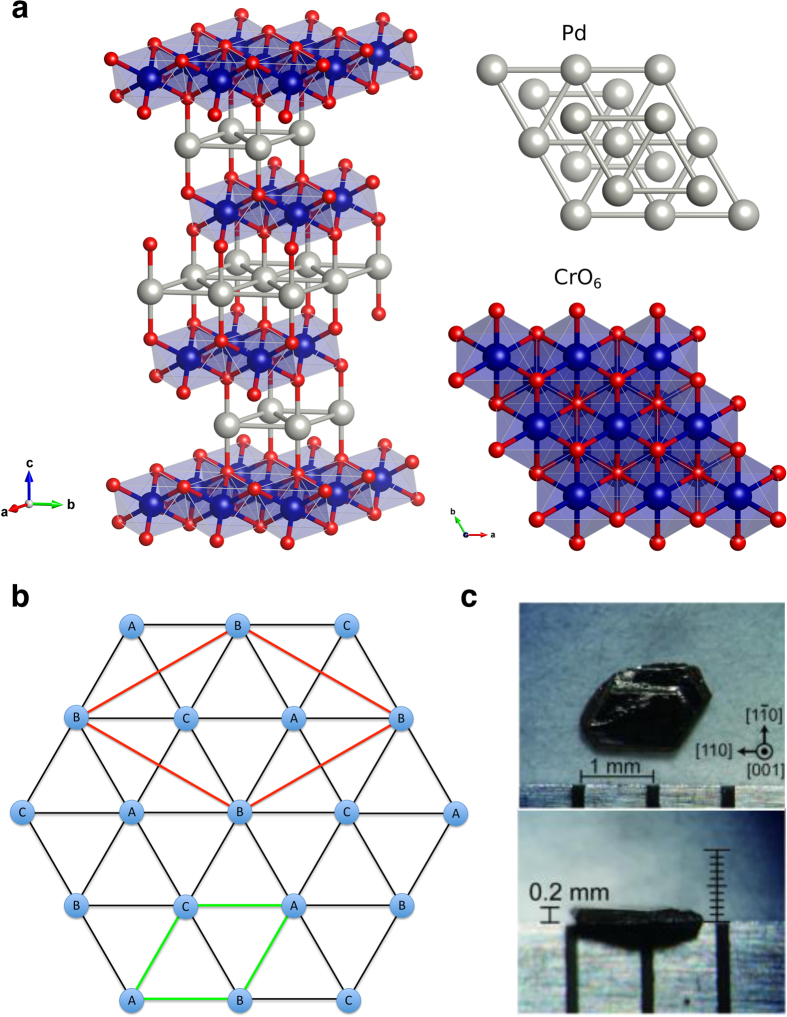
Crystal and magnetic structure of PdCrO_2_. **a**, Crystal structure of the delafossite PdCrO_2_ (space group 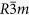). Pd atoms are silver, Cr are blue and O are red. The stacked triangular layers of Pd and CrO_6_ octahedra are also shown. **b**, Diagram of a single triangular layer of Cr atoms from the PdCrO_2_ crystal structure. The three different spin orientations of the Cr magnetic moments are indicated by the letters A, B and C and the crystal (magnetic) unit cell is indicated by the green (red) lines. **c**, PdCrO_2_ single-crystal used in this study.

PdCrO_2_ single-crystals were grown using a NaCl flux method as described in Ref. 11. A mixture of polycrystalline PdCrO_2_ and NaCl with a mass ratio of 1:10 was annealed at 880 °C for 24 hours. This was then cooled to 800 °C at a cooling rate of 0.25–0.50 °Chr^−1^ and then to 700 °C at 1 °Chr^−1^. After this, the crystals were cooled radiatively down to room temperature. Larger crystals were grown by using the smaller crystals as seeds for the next run. The sample used in this experiment was ~1.5 × 1.0 × 0.2 mm^3^ and is shown in Fig. 1c.

### Neutron diffraction

Neutron diffraction measurements were made using the cold neutron WISH time-of-flight diffractometer at the ISIS facility of the Rutherford Appleton Laboratory, United Kingdom^33^. WISH is equipped with two continuous arrays of ^3^He detectors with in-plane angular coverage of 10° < ±2*θ* < 170° and ±15° out-of-plane. This provides the substantial **Q**-space coverage required for single-crystal experiments. The WISH instrument resolution and flux can be tuned depending on the experimental requirements.

At *T* = 150 K, the intrinsic resolution was Δ*d*/*d* = 0.8%. From the size of the non-magnetic Bragg peaks, we estimate that the mosaic of a single crystallite contributes an angular radius of approximately 1.3°. The peak flux on WISH is at a neutron wavelength of 3.5 Å and the crystal was aligned in order to have the magnetic Bragg peaks as close to this as possible.

**Figure 2:**
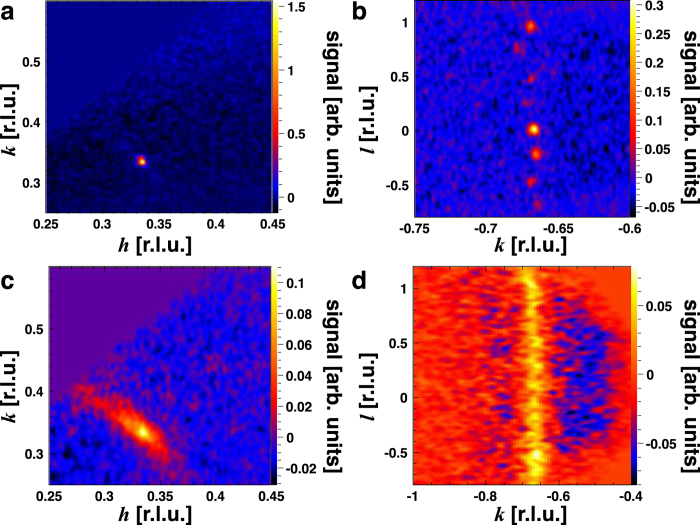
Neutron diffraction from PdCrO_2_ above and below the ordering temperature. **a**,**b**, Magnetic scattering at *T* = 35.0 K in the (*hk*0) and 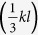 planes, respectively. The areas of high intensity indicate the magnetic Bragg peaks which are resolution limited in the *hk*-plane but broader along *l* implying that, even in the ordered phase, the system is intrinsically less ordered along the crystallographic *c*-axis. **c**,**d**, Magnetic scattering at *T* = 38.5 K in the (*hk*0) and 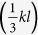 planes, respectively. The higher intensity regions centred around the location of the magnetic Bragg peaks in the *hk*-plane indicate diffuse scattering from short-range antiferromagnetic spin correlations. In the *hk*-plane, the diffuse scattering is quite broad, whilst along *l*, the diffuse scattering develops into continuous rods indicating that, along the crystallographic *c*-axis, the magnetic moments are only weakly correlated.

**Figure 3:**
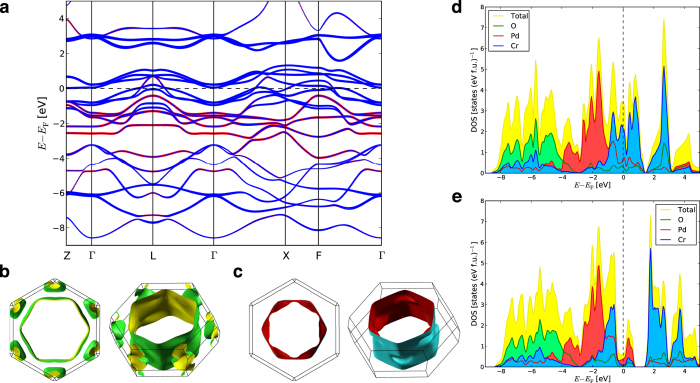
Calculated electronic structure of PdCrO_2_. **a**, Calculated band structure of paramagnetic PdCrO_2_ along selected high-symmetry directions. The thickness of the band indicates the relative character of Cr *d*-electron (blue) plotted over the Pd *d*-electron (red). **b**, Band 1 Fermi surface sheet viewed down the c^*^-axis (left) and a low-symmetry direction (right). **c**, Band 2 Fermi surface sheet viewed down the c^*^-axis (left) and a low-symmetry direction (right). **d**, Calculated DOS of PdCrO_2_ in the paramagnetic phase, showing the total DOS in yellow (upper curve), the site-projected Pd DOS in red, Cr DOS in blue and O DOS in green. The interstitial DOS is not plotted. **e**, Calculated DOS of PdCrO_2_ in the non-collinear antiferromagnetic phase. The colours are the same as in **d**. The interstitial DOS is not plotted.

**Figure 4:**
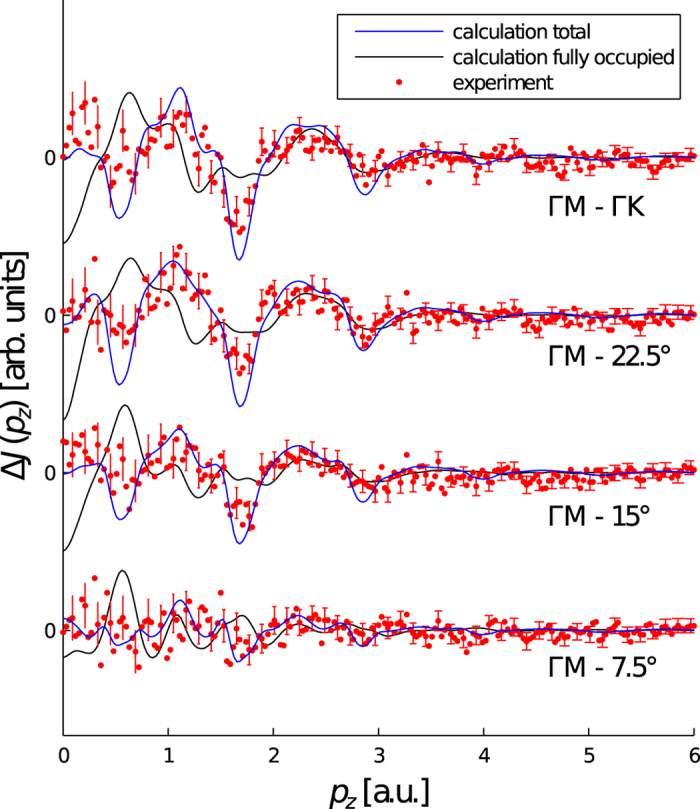
Directional differences of the experimental and calculated Compton profiles. Directional differences, Δ*J*(*p*_*z*_), of the experimental (red dots) and calculated Compton profiles for all of the bands (blue line) and only the fully occupied bands (black line). The profiles are spaced equally between the Γ-M and Γ-K directions of the hexagonal Brillouin zone and the angle is measured from the Γ-M direction. The calculation has been convoluted with a one-dimensional Gaussian approximating the experimental resolution function. The error bars are plotted for every fourth point and indicate statistical errors of one standard deviation.

**Figure 5:**
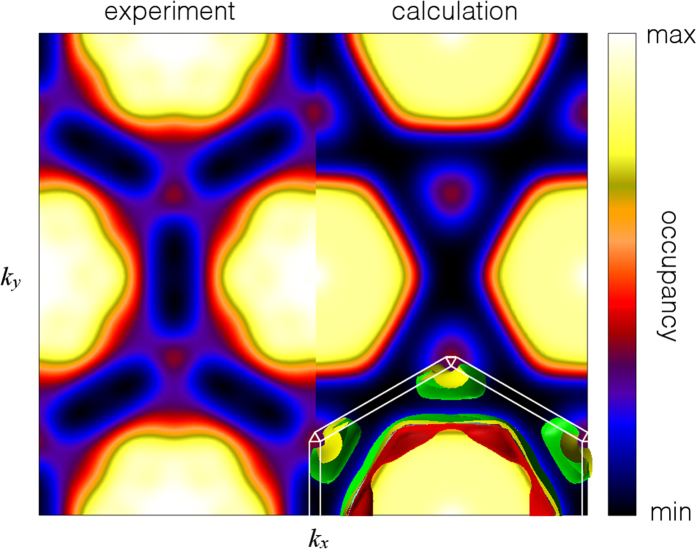
Projected occupation density of PdCrO_2_. Experimental (left) and calculated (right) occupation density of PdCrO_2_ projected down the c^*^-axis. The experiment was performed at *T* = 298 K. The calculated occupation density has been convoluted with a two-dimensional Gaussian approximating the experimental resolution function. The two calculated Fermi surface sheets are superimposed in the lower right quadrant to illustrate where features in the projection originate. The outer (band 1) and inner (band 2) Fermi surface sheets are coloured green and red, respectively, and the thin white lines indicate the rhombohedral Brillouin zone boundary.

**Figure 6:**
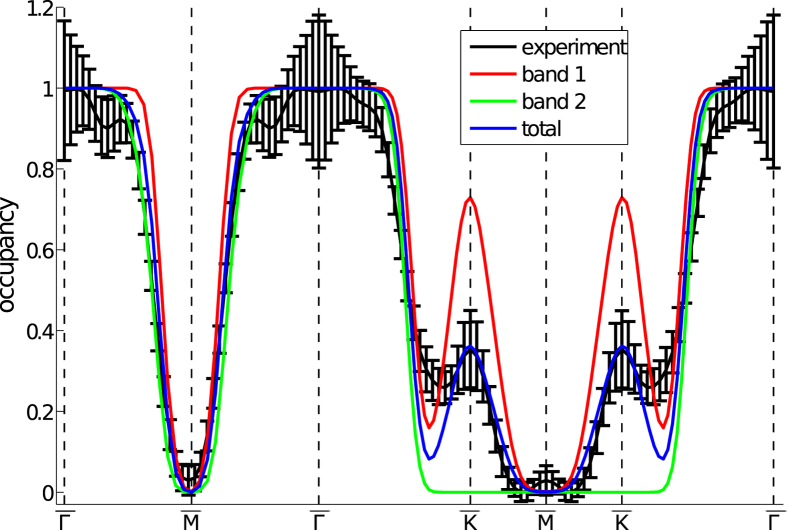
Path through the projected occupation density of PdCrO_2_. The occupation density from the experiment is plotted along the projected high symmetry directions. For comparison, the densities predicted by the electronic structure calculation decomposed into the contributions of each band (and their sum) are also plotted, in each case normalised to unity at the zone centre. The projected occupation density was convoluted with a two-dimensional Gaussian approximating the experimental resolution function before the path was taken. The error bars indicate a statistical error of one standard deviation.

**Figure 7:**
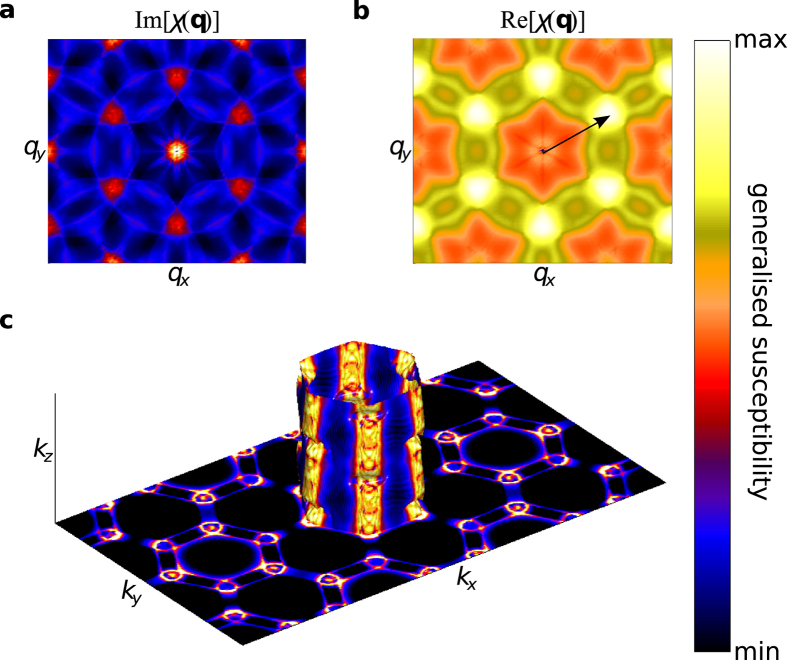
Generalised susceptibility of PdCrO_2_. **a**, Imaginary and **b**, real parts of the noninteracting susceptibility (Eq. (3)), *χ*_0_(**q**), for band 2 intraband transitions in the **q** = (*q*_*x*_, *q*_*y*_, 0) plane. The arrow indicates the nesting vector 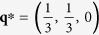. **c**, Real part of the **k**-resolved noninteracting susceptibility (Eq.(4)), *χ*_0,**q**_(**k**), of band 2 for **q** = q^*^. 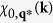 is plotted in the **k** = (*k*_*x*_, *k*_*y*_, 0) plane and on the Fermi surface of band 2. The colours in the plane indicate the value of 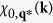, and the colours on the Fermi surface indicate the value of 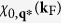. Lighter (darker) colours indicate a large (small) susceptibility.

The diffuse magnetic scattering was isolated by subtracting a high temperature (*T* = 150 K) background from the *T* = 38.5 K data. The subtraction results in the negative scattering intensity seen in Fig. 2. The shape of the scattering seen in the *hk*-plane may (in part) arise from the diffractometer integrating over low lying magnetic excitations that persist in the system above *T*_N_. The scattering function measured on WISH, *S*(**Q**), is the integration of the dynamic scattering function, *S*(**Q**, *ω*), over a 45 meV bandwidth (*S*(**Q**, *ω*) accounts for all of the scattering, both elastic and inelastic).

The correlation lengths were determined by fitting the sharp magnetic Bragg peaks and broader regions of diffuse magnetic scattering with Voigt functions and extracting the full-width-at-half-maximum (FWHM). The correlation length is then given by^34^,


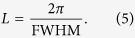


### Computational details

The ELK code^35^, a highly accurate all-electron full-potential augmented plane-wave plus local orbital (FP-APW + lo) method, was used to determine the ground-state electronic structure. PdCrO_2_ crystallises in the delafossite structure (space group 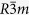, Pd at (0, 0, 0), Cr at 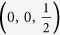 and O at (0, 0, ±*z*_o_), see Fig. 1a)^11^. The lattice constants and internal oxygen coordinate were fixed at the experimental values (*a* = 2.9228 Å, *c* = 18.093 Å, *z*_o_ = 0.1105)^14^ and calculations were made with a cutoff for plane-waves (in the interstitial region) determined by 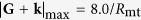, where *R*_mt_ is the average muffin-tin radius. The muffin-tin radii for Pd, Cr and O were 2.2037 a.u., 2.2460 a.u. and 1.4277 a.u., respectively. Convergence was obtained on a 32 × 32 × 16 **k**-point mesh giving 2601 **k**-points in the irreducible Brillouin zone. For the exchange-correlation functional, the Perdew-Burke-Ernzerhof^36^ generalised gradient approximation (PBE-GGA) was used. Spin-orbit coupling was included in the calculation by adding a term of the form ***σ***** · L**, where ***σ*** is the spin vector and **L** is the orbital angular momentum vector, to the second variational Hamiltonian. The calculated electron momentum densities and Compton profiles were produced from the calculated electronic structure by the method of Ernsting *et al.*^37^.

### Compton scattering

The measurements were performed at room temperature (*T* = 298 K) on the high-resolution x-ray Compton spectrometer of beamline BL08W at the SPring-8 synchrotron, Japan^38^. The incident x-ray energy was 115 keV and the scattering angle was 165°. Each Compton profile had approximately 10^5^ counts in the Compton peak and a full-width-at-half-maximum resolution of 0.106 a.u.. Each Compton profile was corrected for absorption, analyser and detector efficiencies, scattering cross-section, double scattering contributions and background. The core electron contributions were then subtracted from each profile.

### Data Accessibility

The underlying research materials can be accessed at the following DOI: 10.5523/bris.l44seqw1wb6d1rxtsscnicvgw.

## Additional Information

**How to cite this article**: Billington, D. *et al.* Magnetic frustration, short-range correlations and the role of the paramagnetic Fermi surface of PdCrO_2_. *Sci. Rep.* **5**, 12428; doi: 10.1038/srep12428 (2015).
